# Prolonged duration induces divergent transcriptomic responses to manganese, distinct from concentration effects, in an SH-SY5Y neurotoxicity model

**DOI:** 10.1016/j.neuro.2026.103393

**Published:** 2026-01-24

**Authors:** Xueqi Tang, Priyanka Baloni, Michael Aschner, Aaron B. Bowman

**Affiliations:** a School of Health Sciences, Purdue University, West Lafayette IN, United States; b Dept of Molecular Pharmacology, Albert Einstein College of Medicine, Bronx NY, United States

**Keywords:** Manganese neurotoxicity, Chronic exposure, Transcriptomics, SH-SY5Y cells

## Abstract

Understanding manganese (Mn) neurotoxicity requires experimental models that realistically reflect human exposure scenarios. A key limitation of current *in vitro* paradigms is the reliance on acute, high-concentration exposures, which may not accurately capture the molecular consequences of long-term Mn accumulation. To address this, this study compared transcriptomic responses to acute (6-hour) and chronic (40-day) Mn exposures in SH-SY5Y cells, using Mn concentrations spanning near-physiological to sub-cytotoxic ranges. The 6-hour exposure design replicates a widely applied acute duration in the literature, while the 40-day duration was selected to mimic prolonged, low-level Mn burden reported in epidemiological and occupational studies. Bulk RNA sequencing revealed that chronic Mn exposure induced distinct and more extensive transcriptional alterations compared to acute exposure, independent of concentration. Pathway enrichment analyses indicated that cellular functions selectively perturbed under chronic conditions are highly relevant to neurodegenerative risks and aligns with independent Parkinson’s disease transcriptomic datasets. These pathways include axonal guidance signaling, amyloid fiber formation, extracellular matrix organization, and synaptic functioning. In contrast, acute exposures primarily disturbed intracellular ion homeostasis maintenance mechanisms. Protein kinase A signaling and metallothionein-mediated metal-binding pathway were the only two pathways that were shared between both applied durations exposed at Mn concentrations with reported adverse outcomes. Transcriptomic alterations in this study highlighted the contribution of mechanisms related to normal Mn-dependent cellular functions in the development of its neurotoxicity. Furthermore, these results emphasized that exposure duration is a critical determinant to be considered when evaluating long-term Mn overload-induced neurodegeneration via *in vitro* platforms.

## 1. Introduction

Since the first report of Mn-induced neurotoxicity, efforts have been made to replicate brain Mn overload in experimental models aiming to unravel its mechanisms. The wide time and concentration ranges of Mn associated with adverse neurological and neuropsychological outcomes, together with the lack of valid biomarkers for quantifying post-exposure intracellular Mn levels have created obstacles in developing translational models for mechanistic studies, especially for cell-based experimental design (Fernández-Olmo et al., 2020; Karyakina et al., 2022; Mehrifar et al., 2020). Animal models capturing Mn-induced behavioral dysfunctions led earlier investigations and revealed mechanisms including but not limited to oxidative stress, mitochondrial dysfunction, endoplasmic reticulum stress, inflammation, apoptosis and autophagy disruptions (Cheng et al., 2023; Harischandra et al., 2019; Tinkov et al., 2021). Leveraging insights from animal modeling to *in vitro* experimental design, the exposure concentrations were commonly determined by estimating the post-exposure Mn elevation and identifying LC_50_ concentration dose-dependent viability curve. This rationale yielded a range of 100 μM – 1 mM Mn which recapitulated molecular features of *in vivo* neurotoxicity within hours of exposure, and therefore led to a working premise that acute *in vitro* exposures at high concentrations could reflect and predict Mn neurotoxicity mechanisms.

However, challenges have arisen against the translational power of acute high concentration exposure *in vitro* models. With regulatory actions taken to address the Mn exposure-associated health risks, workplace Mn exposure levels have been largely decreased accompanied by increasing concerns for lifelong exposures due to air and drinking water contamination (Lucchini et al., 2009). Therefore, a shift of scope has occurred to further decipher the risk of extended exposures without inducing immediate cytotoxicity. Furthermore, in-depth investigations have distinguished Mn-induced Parkinsonian-like symptoms from sporadic Parkinson’s disease and confirmed neurodegenerative risks associated with chronic Mn overload that do not invoke significant acute neuronal death (Kwakye et al., 2015; Martins et al., 2020). The need to reduce exposure concentrations and extend exposure durations to better model real world chronic exposures is also evoked by reported discordance of cellular responses to different exposure paradigms. Reduced exposure levels have displayed distinct impacts compared to high concentrations in both cell- and animal-based models. In rats, 200 mg/kg body weight MnO_2_ nanoparticle inhalation resulted in distinguishable behavioral changes and reversed choroid plexus transcriptomic changes compared to exposure at 400 mg/kg (Meng et al., 2023). In cultured SH-SY5Y neuroblastoma cell line, Fernandes et al. reported that with the increase of Mn concentration, key transcriptomic responses shifted from protein secretion to oxidative phosphorylation in acute exposures (Fernandes et al., 2019). Similarly, extensions of the exposure duration altered the outcomes. One, three-, and five-day exposures led to time-dependent increases of number of differentially expressed genes (DEGs) detected in rat liver with less than 10 % overlap (B. Yang et al., 2023). In cultured immortalized striatal neuron model, 30-hour non-cytotoxic Mn exposure at 50 μM was reported to differently impact the AKT/S6 signaling pathway compared to 6-hour exposures. Specifically, augmentation of the upstream AKT phosphorylation declined with the extension of the exposure, while downstream S6 phosphorylation remained elevated (Tang et al., 2023). Divergent effects on cellular signaling was not only observed in *in vitro* models. A significant decrease of neurogenesis regulatory gene *EOMES* and its encoded protein Tbr2 was observed only in the mouse subgranular zone when animals were exposed to Mn orally for 56 days, but not at the 28-day intravenous injected animals despite more cerebral Mn accumulation (Kikuchihara et al., 2015). These studies suggest a dissimilarity between neurotoxicity induced by different paradigms and argue against the premise that acute cytotoxic exposures predict mechanisms of extended near-threshold Mn concentrations. To date, no comprehensive study has directly compared the impact of exposure level and duration in totality with cell-based models. To fill this knowledge gap, this study was designed to compare transcriptomic response to chronic and acute exposures at multiple non-cytotoxic Mn concentrations. Differentiated human neuroblastoma line SH-SY5Y was employed as the modeling system considering its wide application in the field and bridging translational power between immortalized cell line and human stem cell-derived neuronal models. We aimed to test the hypothesis that beyond the discordant cellular response to near-physiological versus toxic Mn concentrations, acute and chronic Mn affect neuronal functions via distinct mechanisms as well.

## 2. Method

### 2.1. Cell culture

**Fig. 1.**
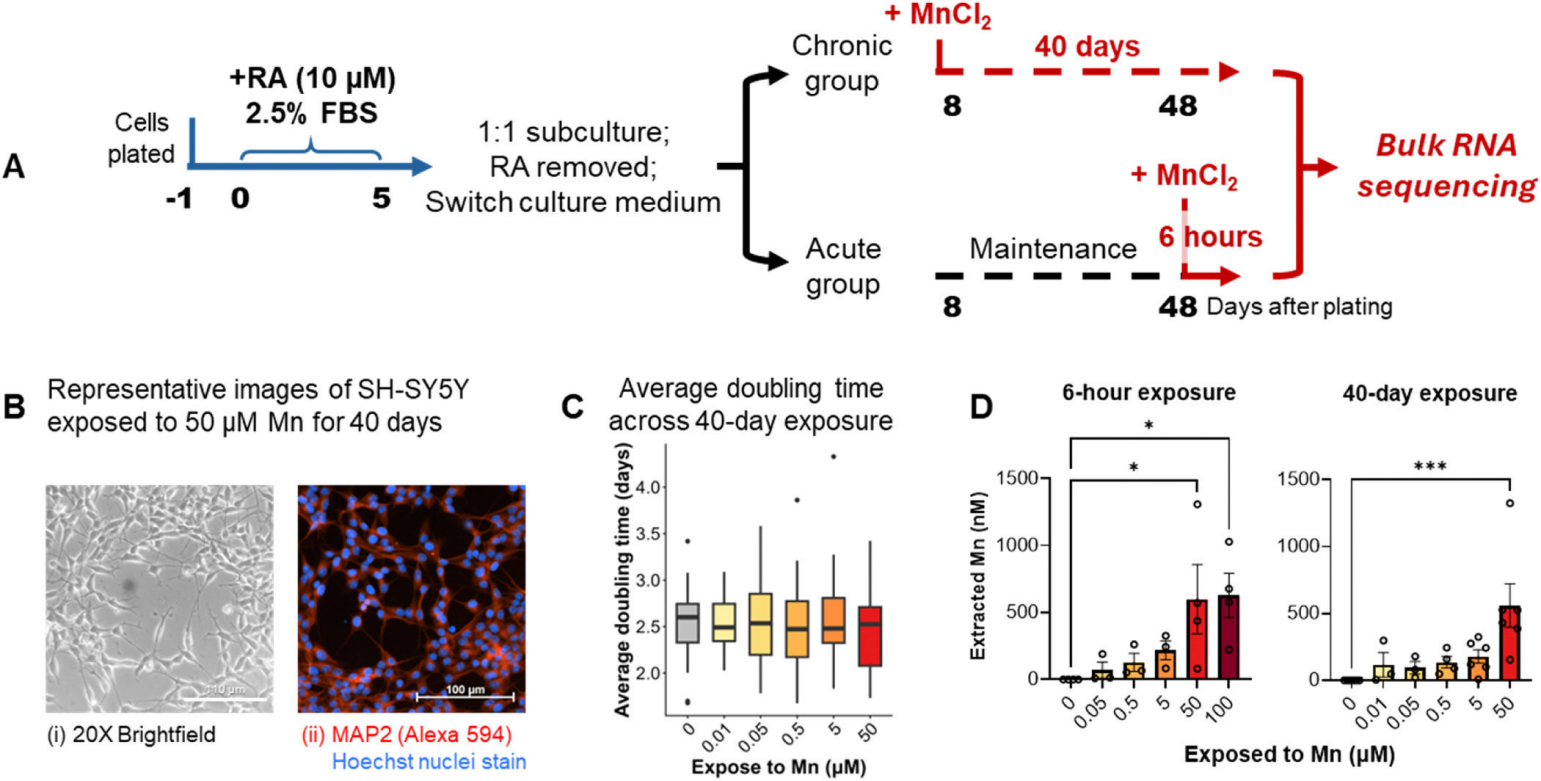
Experimental design and phenotypical evaluations. (A) Differentiation of SH-SY5Y neuroblastoma line and exposure design. RA, retinoic acid. (B) Representative images of SH-SY5Y exposed to 50 μM Mn for 40 days. (i) Bright field and (ii) immunofluorescence image of microtubule-associated protein 2 (MAP2, Alexa 594, red) and nuclei (blue). (C) Average doubling time in the 40-day exposure group during the exposure phase. (D) Intracellular Mn by the end of the 6-hour (left) and 40-day (right) exposure measured by cellular Fura-2 manganese extraction assay (CFMEA). * *P* < 0.05, *** *P* < 0.0001 determined by ordinary one-way ANOVA followed by Dunnett’s multiple comparison test.

The SH-SY5Y cell line was purchased from American Type Culture Collection (ATCC, CRL-2266) and cultured with Dulbecco’s Modified Eagle Medium/Nutrient Mixture F-12 with GlutaMax supplement (DMEM/F-12, GlutaMax, Gibco, Cat. #: 10565018) with 10 % fetal bovine serum (FBS, R&D Systems, Cat. #: S11150) and 1 % penicillin-streptomycin (Gibco, Cat. #: 15140122) at 37°C in a humidified atmosphere containing 5 % CO_2_. The expanded culture was replated into 6-well plates for differentiation and exposure as illustrated in Fig. 1A. The replated cells first went through 10 μM all-trans retinoic acid (RA, CAS 302-79-4, Thermo Scientific Chemicals, Cat. #: AC207341000) differentiation for 5 days in DMEM/F-12 with GlutaMax supplied with 2.5 % FBS (Shipley et al., 2016).

On day 5, differentiated SH-SY5Y cells displayed outgrowth of neuronal projections compared to cell morphology before RA treatment and without RA treatment (Fig. 1B), and were subcultured into 6-well plates and divided into two groups for further maintenance and exposures. In the meantime, the culture medium was switched to human induced pluripotent stem cell (hiPSC)-derived cortical neuron maintenance medium (abbreviated as CNM) composed of 1:1 mixture of B27 neurobasal medium (Neurobasal medium (Gibco, Cat. #: 21103049) supplied with 1X B27 (Gibco, Cat. #: 17504044) and 1X GlutaMax (Gibco, Cat. #: 35050061)) and N-2 DMEM/F-12 medium (DMEM/F-12 with GlutaMax supplied with 1X N-2 (Gibco, Cat. #: 17502048), 1X MEM non-essential amino acid solution (Gibco, Cat. #: 11140050), 2 % penicillin-streptomycin, and 100 μM 2-mercaptoethanol (CAS 60-24-2, Sigma-Aldrich, Cat. #: M3148) (Shi et al., 2012). The purpose of switching to CNM is (1) to supply the culture with neurotrophic factors to maintain the neuron-like phenotype while excluding the impact on signaling pathways that can be introduced by RA treatment; and (2) to match the culture and exposure environment with cortical cultures derived from hiPSCs in order to control for any implication that the supplementation system may have on cellular response to toxicants, which has been reported earlier in a comparison of toxicant vulnerability across different neuronal lineages (Joshi et al., 2019). A compatible culture system between SH-SY5Y and hiPSC-derived cortical cultures would allow future comparisons aiming to feed into the development of comprehensive *in vitro* modeling of Mn neurotoxicity.

Following 3 days of adaptation to the CNM, the 40-day chronic exposure group started to receive Mn treatment by adding 0, 0.01, 0.05, 0.5, 5, and 50 μM MnCl_2_ to the culture medium. The 6-hour acute exposure group was maintained with CNM till 6 hours before harvest, when the Mn exposure was applied at 0, 0.05, 0.5, 5, 50, and 100 μM. The lower end of exposed concentrations was selected based on the physiological Mn content in human cerebrospinal fluid (CSF, estimated around 0.05 μM) and in widely used 10 % v/v FBS supplied cell culture media (ranging from 0.05 to 0.12 μM) (Cho et al., 2007; Oulhote et al., 2014; Vicogne et al., 2020). A concentration that was lower than the estimated physiological level, 0.01 μM, was included considering the complicated distribution of Mn in extracellular spaces in human brain and the difference of Mn speciation between *in vivo* and *in vitro* systems. The higher end was determined based on reported Mn dose-dependent cell viability curve and acute no observed adverse effect level (NOAEL) concentration of Mn in SH-SY5Y cells (50 μM) (Fernandes et al., 2017; Gandhi et al., 2018; Hernandez et al., 2021). Each treatment group has an N of 5.

### 2.2. Cellular Fura-2 Manganese Extraction Assay (CFMEA)

CFMEA assay was performed for intracellular Mn content quantification as described earlier (Kwakye et al., 2011). In brief, the cells were seeded in 96-well plates 48 hours prior to the end of the Mn exposure. By the end of the Mn treatment, cells were lysed in PBS with 0.1 % Triton X-100 containing 0.5 μM Fura-2. The cells were incubated with Fura-2 containing lysis buffer for 10 min to allow complete extraction of intracellular Mn content and quenching of Mn ion with the Fura-2 compound. The Fura-2 fluorescence was measured at Ex_360_/Em_535-nm_, and total extracted intracellular Mn calculated through an established cell-free Mn-Fura-2 standard curve.

### 2.3. RNA isolation and mRNA sequencing library preparation

By the end of the exposure, cells were collected with lysis buffer from Invitrogen PureLink RNA mini kit (Invitrogen, Cat. #: 12183018 A), processed with QIAshredder mini spin column (QIAGEN, Cat. #: 175028900), and RNA isolated with the PureLink RNA mini kit following manufacturer’s instructions. Isolated RNA was quantified by Nanodrop One (Thermo Scientific) while confirming A260/A280 greater than 1.8 and integrity measured by RNA ScreenTape (Agilent) greater than 9.3. mRNA sequencing library was prepared using Tecan Universal Plus mRNA sequencing library preparation kit (Tecan, Cat. #: 5020) following manufacturer’s instructions. Quality control and pooling of the library was performed by the Indiana University Medical Genomics Core. The pooled library was sequenced with the Illumina NovaSeq X plus system yielding an average of ~20 M reads/sample.

### 2.4. Bioinformatics and statistical analyses

Reads quality was evaluated with FastQC Version 0.11.9 (https://www.bioinformatics.babraham.ac.uk/projects/fastqc/) and MultiQC (Ewels et al., 2016) showing an overall per sequence quality score (Phred score) between 35 – 36. Trimming was not performed considering the minimal content of adapter sequences (less than 2 %). The reads were aligned to the hg38 human reference genome (https://hgdownload.soe.ucsc.edu/goldenPath/hg38/bigZips/latest/hg38.fa.gz) with STAR v2.7.10a (Dobin et al., 2013) and quality checked with FastQC. Aligned reads were mapped and summarized with featureCounts to generate gene-level count matrices (Liao et al., 2014). Following the alignment and mapping, batch effects were adjusted with the ComBat-seq package and differentially expressed genes (DEGs) were determined by DESeq2 (Love et al., 2014; Zhang et al., 2020).

Logarithmic fold change in DESeq2 is estimated by employing an empirical Bayes procedure, where generalized linear model is fitted on a per-gene basis to obtain maximum-likelihood estimates. Output of the logarithmic fold change, reported as log2FC, is interpreted as the effect size. For the hypothesis testing, DESeq2 uses the Wald test. *P* values from the Wald test are then adjusted for multiple testing using Benjamini-Hochberg procedure and reported as adjusted *P* values (padj) and interpreted as false discovery rate. For the chronic group, genes that displayed a fold change greater than 1.5 (log2FC > 0.58) and a padj less than 0.1 were considered as differentially expressed. For the acute group, considering the limited expression changes induced by the exposure, genes that displayed a padj less than 0.1 were considered as differentially expressed.

Following the acquisition of DEG lists, enrichment analysis was performed to understand whether a predefined group of genes were impacted by the exposures. Hypergeometric test followed by Benjamini-Hochberg method for false discovery rate correction in multiple testing is employed in the analyses with an output of adjusted *P* values reported and interpreted as significance levels. Further details of the statistical methods were described in the figure legends where appropriate.

### 2.5. Publicly available datasets

Aligned counts were acquired from GSE206308 and differential expression analysis was performed with DESeq2 as indicated by Verma et al. (2023). This dataset was derived from human brain substantia nigra pars compacta (SNpc) from control individuals and PD patients. The other dataset was acquired from DEGs reported by Fiore et al. contrasting RA differentiated SH-SY5Y treated with PD-related neurotoxin 1-methyl-4-phenylpyridinium (MPP) and non-treated control (Fiore et al., 2022). DEGs reported in both datasets, in combination within the present study were filtered by cutoff of baseMean > 10 and padj < 0.1 and merged for further analysis. Pathway analysis was performed with ShinyGO 0.82 (Ge et al., 2020).

## 3. Results

### 3.1. No cytotoxicity was observed during either acute or chronic dosing of Mn at concentrations that were applied

The SH-SY5Y cells first underwent RA-induced differentiation that yielded significant neurite outgrowth compared to cultures that did not receive RA treatment (Supp. Figure 1). Differentiated SH-SY5Y cells were then subcultured for CNM conditioning and Mn exposures. By the end of the Mn treatment, the neurites were partially maintained by the N2 and B27 supplements in the CNM, with no significant morphological deficiency observed either by brightfield microscopy or immunofluorescent staining of neuronal marker microtubule-associated protein 2 (MAP2) up to 50 μM Mn (Fig. 1B). During the chronic exposure, cell counts at each subculture timepoint were recorded and a doubling time (DT) was calculated by the formula DT = Cell culture time/Cell number (N) at each Mn treatment concentration. In the formula, cell culture time reflected the days between each passage and cell number was calculated by N = ln(N_harvest_/N_plated_)/ln2, where N_harvest_ was the number of cells harvested counted with Cellometer (Nexcelom, Cellometer Auto T4) at each subculture time point and N_plated_ was the number of cells plated (Lee et al., 2013). During the Mn-exposure phase, SH-SY5Y cells displayed an average doubling time of 3–4 days, which is higher than the reported 27–52 hours in non-differentiated SH-SY5Y line, suggesting a successful inhibition on the proliferation by the initial RA treatment and the adaptation to CNM (Kovalevich and Langford, 2013). None of the Mn concentrations had a significant impact on the doubling time of the SH-SY5Y culture over the 40-day exposure time (Fig. 1C).

Total intracellular Mn content was measured by CFMEA by the end of both the 6-hour and the 40-day exposure (Fig. 1D). CFMEA is a fast measurement of total intracellular Mn content by Fura-2 fluorescence at Ex_360 nm_ with an accurate detection limit of 100 nM Mn (Kumar et al., 2013). 50 μM Mn and beyond induced significant elevation of intracellular Mn content in both 40-day and 6-hour exposures. Combined with reported neurotoxicity in SH-SY5Y cells, concentrations beyond 50 μM were considered as high toxic levels in the following analysis (Gandhi et al., 2018; Hernandez et al., 2021). 0.5 and 5 μM exposure for both 40-day and 6-hour, although did not induce statistically significant increase detected by quenched Fura-2 fluorescence as per CFMEA method, but elevated intracellular Mn content to over 100 nM as compared to below detection limit in non-exposed group. Therefore 0.5 and 5 μM was considered as exposure levels that induced moderate elevation. Mn concentrations at 0.05 μM and below, based on the estimated human CSF Mn level and detected Mn in FBS cell culture supplement, was proceeded as near-physiological concentrations (Cho et al., 2007; Oulhote et al., 2014; Vicogne et al., 2020). Taking these results together, we confirmed that the Mn exposure concentrations and time frames that were applied in this study created an exposure model that significantly increased intracellular Mn concentrations without inducing immediate morphological or growth rate loss.

### 3.2. Levels of Mn in 40-day exposures had a substantial impact on the pattern and magnitude of changes to signaling pathways associated with the differentially expressed genes (DEGs)

**Fig. 2.**
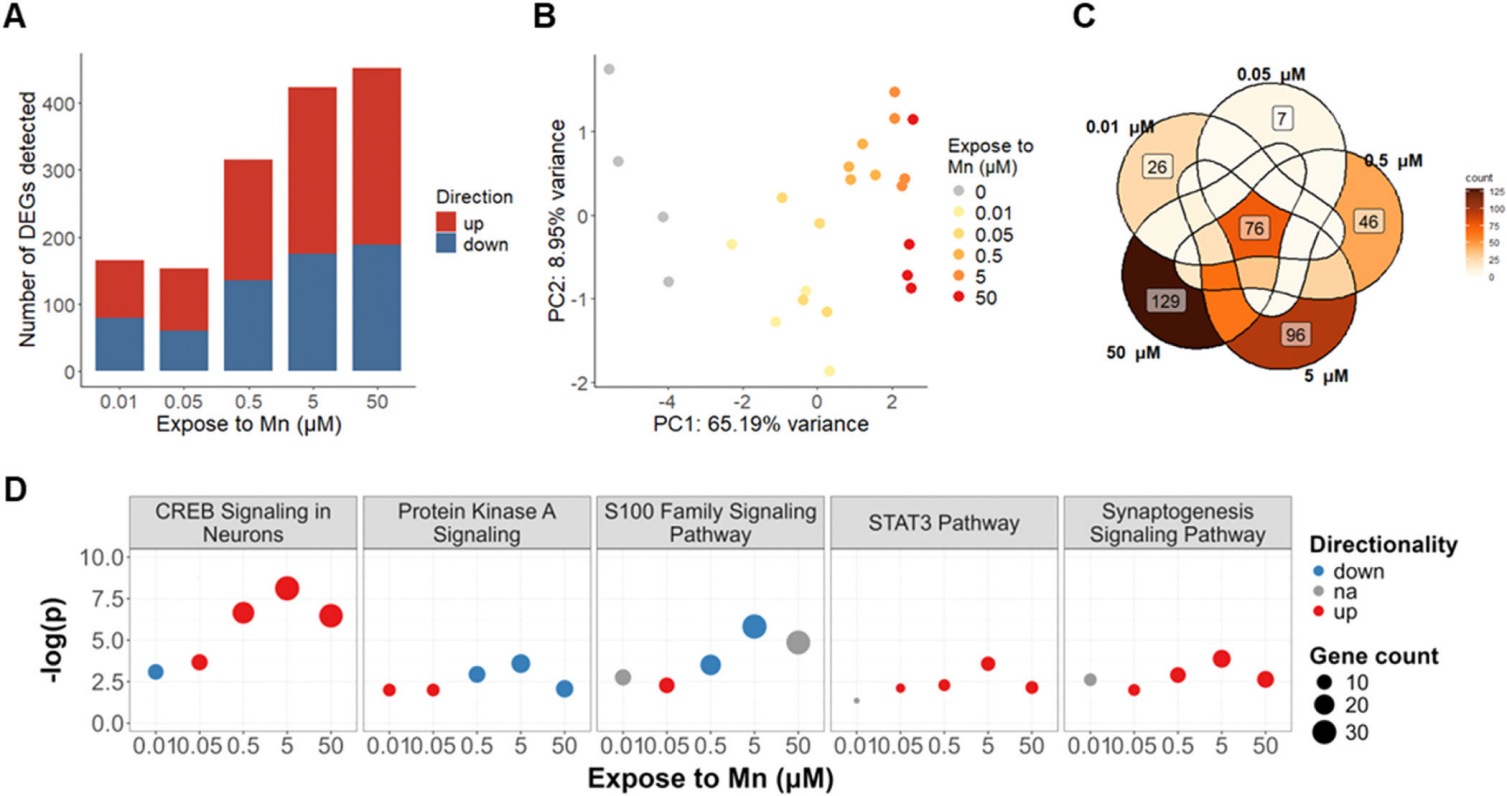
Differentially expressed gene (DEG) detection and Qiagen Ingenuity Pathway Analysis (IPA) in 40-day exposed group. (A) Number of DEGs detected by a cutoff of fold change > 1.5 and adjusted p value < 0.1. (B) Principal component analysis (PCA) plot of variance in gene counts of the 40-day exposure group. (C) Venn diagram visualization of detected DEG overlapping across exposed Mn concentrations. (D) Significance level represented by -log(p-value) detected by IPA in selected pathways of interest. Directionality was determined based on IPA calculated z-scores, where a score ≥ 2 predicts an upregulation (red) and score ≤ 2 predicts a downregulation (blue) of the pathway. A ‘na’ in directionality indicates that there were no sufficient number of genes associated with a particular pathway to provide reliable predictions.

As described in the method section, following pre-processing of the aligned reads, DEGs were determined with DESeq2 by contrasting each Mn concentration to non-exposed control (Love et al., 2014; Zhang et al., 2020). For the chronic group, genes that displayed a fold change greater than 1.5 and a false discovery rate (determined by adjusted p value for multiple testing using Benjamini-Hochberg method) less than 0.1 were considered as differentially expressed. For the acute group, considering the limited expression changes induced by the exposure, genes that displayed a false discovery rate of less than 0.1 with no fold change cutoff were considered as differentially expressed. There were substantially more DEGs detected in the 40-day exposure compared to the 6-hour exposure with a Mn concentration-dependent pattern (Fig. 2A, Supplemental Figure 3 A). Principal component analysis (PCA) plot within the chronic exposure group only supports a dose-dependent variance on the PC1 axis, with 0.01 and 0.05 μM clustered closer to non-exposed control, while 0.5, 5, and 50 μM Mn clustered further on the PC1 axis, representing toxic effects (Fig. 2B). The 0.01 μM Mn exposed group, of notice, yielded a slightly higher number of DEGs (165) compared to 0.05 μM (153), with 91 genes shared between these two conditions (Supp. Figure 2 A). This suggested that 0.01 μM Mn treatment for 40-days had specific interferences in SH-SY5Ys that were distinct from 0.05 μM Mn. Gene ontology analysis mapping with the GO molecular functions database suggested that 26 genes that were specifically affected by 0.01 μM Mn were strongly enriched for iron and calcium transportation and ion channel activity (Supp. Figure 2B). Therefore, we postulate gene expression in ion transportation pathways served as an adaptation response to the long-term addition of 0.01 μM Mn in the culture environment while interfering with other divalent metal ion transportation. ICP-MS quantification of the maintenance CNM showed that the culture media contained a negligible level of Mn that was below the detection limit of 182.02 pM (data not shown). Considering the essentiality of Mn, the cell adhesion receptor and growth factor receptor binding pathways that were associated with 0.01 μM Mn treatment may relate to restoration of cellular functions that are dependent on Mn (Supp. Figure 2B).

A Venn diagram, in Fig. 2C, illustrated the number of DEGs that were shared or unique at each Mn concentration. We performed Qiagen Ingenuity Pathway Analysis (IPA) to investigate the cellular functions related to the 76 genes that were shared across all Mn concentrations in the 40-day exposure (Table 1). Extracellular matrix organization, axonal guidance signaling, neuron projection development, and trans-synaptic signaling were detected and are directly associated with neuronal functions. In addition, canonical pathways that were related to kinase signaling, including RAR, PTEN, WNT, and PI3K/AKT/mTOR axis were among the top hits.

To further understand the transcriptomic effects of chronic Mn, IPA was also performed with the full DEG lists acquired at each chronic Mn concentration. We investigated the significance level (represented by -log(p) values, significance cutoff of p < 0.05 (-log(p) > 1.3)) of pathways that were mutually affected yet displayed a high variance across concentrations with the goal of identifying potential dose-dependent effects. Anti-intuitively, the significance levels of multiple pathways did not show a linear significance-dose relationship. Instead, a biphasic pattern with inflection points at 0.05 and 5 μM were observed. Pathways that followed this pattern included CREB signaling, protein kinase A signaling, S100 family signaling, synaptogenesis signaling, and the STAT3 pathway (Fig. 2D). This pattern agreed with the higher number of total and unique DEGs detected in 0.01 μM and again indicated that pathways affected under this condition may represent essential functions that depend on optimal ranges of intracellular Mn levels. With the increase of Mn exposure concentrations, the significance levels increased reflecting adaptation effects. When Mn reached a high toxic concentration (50 μM), the decrease of the significance level of these pathways may reflect a potential failure in responding to excessive Mn overload. This loss in transcriptomic regulation at the chronic 50 μM Mn exposure condition may contribute to neurotoxicity outcomes. DEGs that hit these pathways were also plotted (Supp. Figure 2 C). Trends in the p-values of the signaling pathways were explained by the number of genes that were detected and contributed to those pathways. The concentration-specific expression pattern of these genes has the potential to be identifiers or biomarkers for determining whether a Mn exposure paradigm introduces neurotoxicity in cell-based models.

### 3.3. Genes that drove the variance between acute and chronic exposures were associated with Mn-dependent functions and neurodegeneration.

**Fig. 3.**
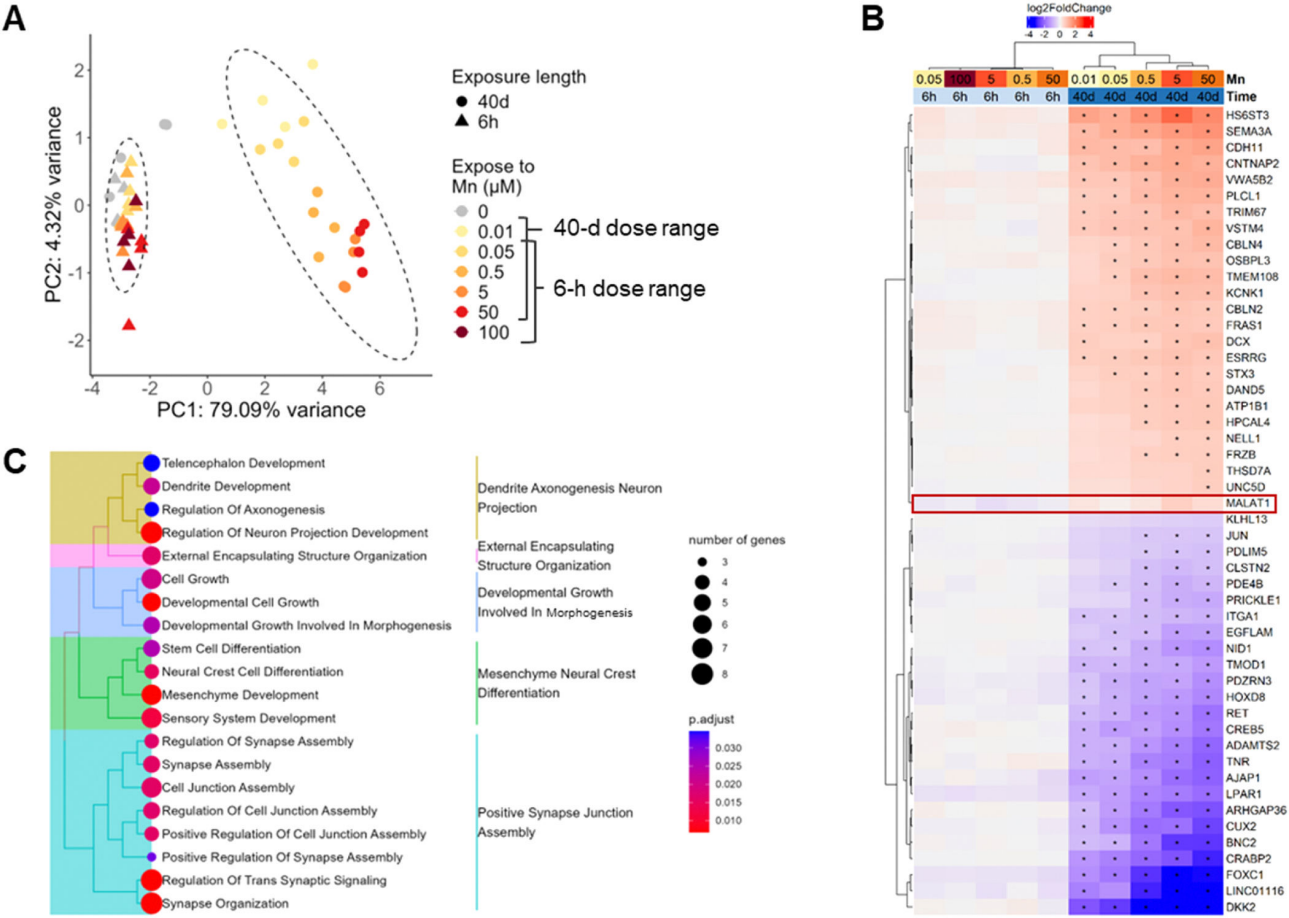
Genes and pathways associated with variance between 6-hour and 40-day exposures. (A) Sample distribution on principal components (PCs) 1 and 2 in principal component analysis (PCA) of gene counts variation across all samples. (B) Heatmap clustering of log2(fold change) of 50 genes with highest absolute loading values in PC1 in Fig. 3A. * Absolute fold change > 1.5 (absolute log2(fold change) > 0.14) and adjusted p value < 0.1. (C) Treeplot of Gene Ontology Biological Processes significantly related to genes listed in Fig. 3B. Significance defined by p value < 0.1 in clusterProfiler enrichment analysis.

Due to the limited number of DEGs detected in 6-hour exposures (Supp. Figure 3 A), we explored the differences between acute and chronic exposure-induced transcriptomic signature via a principal component analysis (PCA) approach. The output showed that all data points from the 6-hour exposure clustered together with the non-exposed data points in both exposure groups on the PC1 axis which explained 79.09 % of the variance, and were set apart from Mn-exposed data points in the 40-day chronic exposure group, suggesting that the length of the exposure explained the vast majority of the variance in gene expression across the total collection of samples under acute and chronic exposures (Fig. 3A). Further, it is noteworthy that as concentration of Mn exposure increased, the distance along PC1 of the acute and chronic samples increased regardless of the acute Mn concentration level.

Fold changes of genes that contributed most to the deviation on PC1, i.e. 50 genes that showed highest absolute loading values for PC1, were plotted in Fig. 3B (full information for each gene listed in Supp. Table 1). Heatmap clustering based on expression patterns revealed that these PC1-contributing genes displayed higher fold changes and significant levels (represented by decreased adjusted p value) under chronic conditions when compared to the acute exposure at corresponding Mn concentration. Enrichment analysis performed by clusterProfiler mapping genes demonstrated in Fig. 3B to GO biological process gene set showed that these genes were associated with axonal and neuronal morphogenesis, stem cell development and differentiation, as well as synapse organization and assembly (Fig. 3C) (Aleksander et al., 2023; Yu et al., 2012). Detection of these pathways further highlighted the impact of chronic Mn exposures on neuronal morphology and synaptic function maintenance whose failures are directly associated with neurodegeneration. Transcriptomic changes in these pathways induced by chronic but not acute exposures suggested that extended exposure duration could better simulate degenerative risks induced by Mn exposures versus the acute 6-hour duration.

In addition to understanding biological processes associated with the differential expression of Fig. 3A PC1-driving genes, we noticed *MALAT1* (metastasis-associated lung adenocarcinoma transcript 1) showed a clear pattern of being downregulated in acute conditions while being upregulated by chronic exposures. Although no statistical significance was detected when comparing *MALAT1* expression in a particular exposure condition to control, significant variance between average fold change in the acute compared to chronic group was detected by two-way ANOVA (Supp. Figure 4). The significantly different average fold change supported that extension of Mn exposures led to distinct transcriptomic alterations.

### 3.4. Mn concentration-dependent gene expression signatures reveal putative toxicity inflection point

**Fig. 4.**
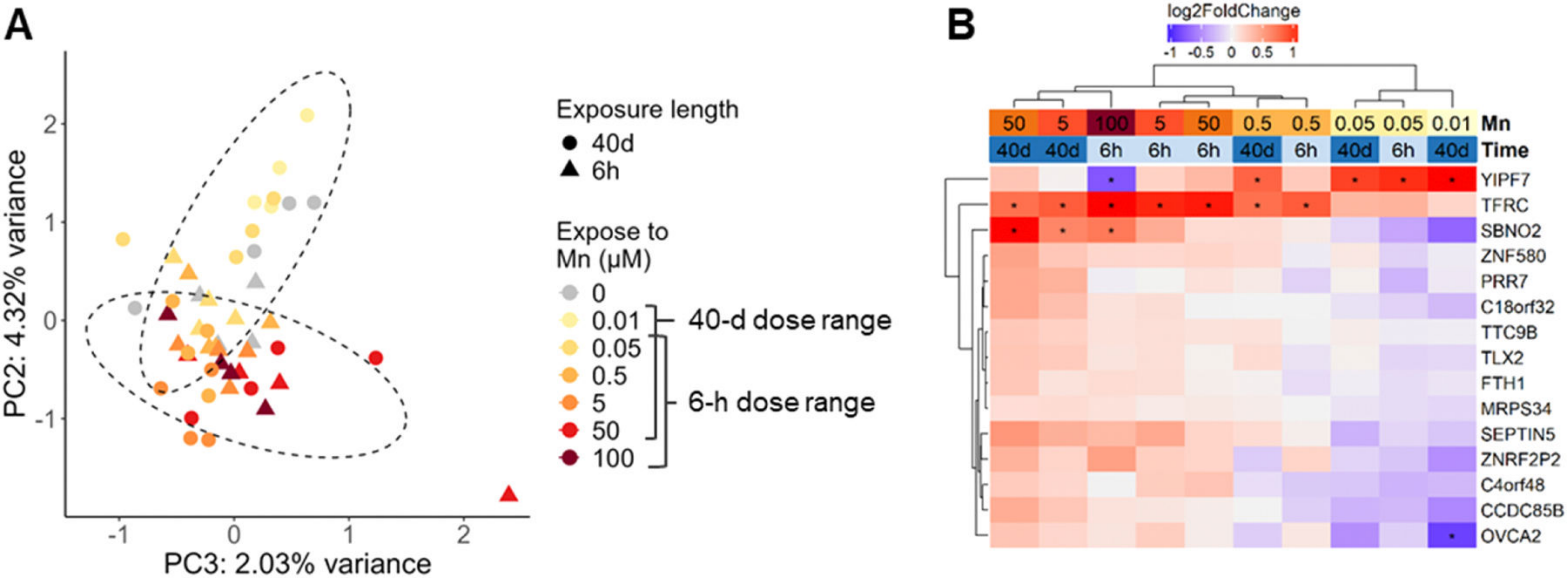
Genes associated with variance driven by increasing Mn concentrations. (A) Sample distribution on principal components (PCs) 2 and 3 in principal component analysis (PCA) of gene counts variation across all samples. (B) Heatmap clustering of log2(fold change) of genes for which variance is mainly attributed to increasing Mn concentrations. * Absolute fold change > 1.5 (absolute log2(fold change) > 0.14) and adjusted p value < 0.1.

In Fig. 3A, it was noted that PC2 displayed a clear pattern of separating samples by Mn concentration independent of the exposure duration pattern of PC1. Therefore, PC2 in Fig. 3A was plotted against PC3 (explaining 2.03 % of the total variance) to explore Mn concentration dependent effects (Fig. 4A). Intriguingly, a butterfly shape of distribution was observed differentiating the distribution of high (50 and 100 μM) from low Mn concentrations (0.01 and 0.05 μM) compared to the control. As performed in the previous section, we again identified the top 50 genes that contributed to the deviation along the Fig. 3A/4 A PC2, followed by variance partition analysis to understand the contribution of exposure levels to the variance at a gene-based scope (Supp. Figure 5) (Hoffman and Schadt, 2016). Combining the list of top 50 genes that contributed to the PC2 separation and genes in which the majority (> 50 %) of their variance was explained by exposure levels, we highlighted a set of 16 genes of which the expression patterns strongly correlated with the increase of Mn concentrations (Fig. 4B, Supp. Table 2). Most of these genes were downregulated by low concentrations of Mn (0.5 μM and below) while upregulated by Mn concentrations close to the toxic range (5 μM and above). The transferrin receptor gene (*TFRC*) was the only gene that was upregulated across all Mn exposure levels. The expression level of *YIPF7*, encoding YIP1 domain family member 7, was significantly enhanced at lower concentrations (0.5 μM and below) while strongly inhibited by 6-hour 100 μM exposure. Expression patterns in this set of genes correlated with the Mn dose-dependent pathway significance changes displayed in 40-day exposures (Fig. 2D). Both results indicate that Mn at 0.5 μM could possibly be an inflection point of Mn’s impact on SH-SY5Y transcriptome. The expression pattern of the selected set of genes that may distinguish sub-toxic from toxic Mn exposure levels possess the potential for differentiating cellular adaptation from toxic response in *in vitro* exposures.

### 3.5. Acute high concentration exposures do not fully predict transcriptomic changes in chronic exposures

**Fig. 5.**
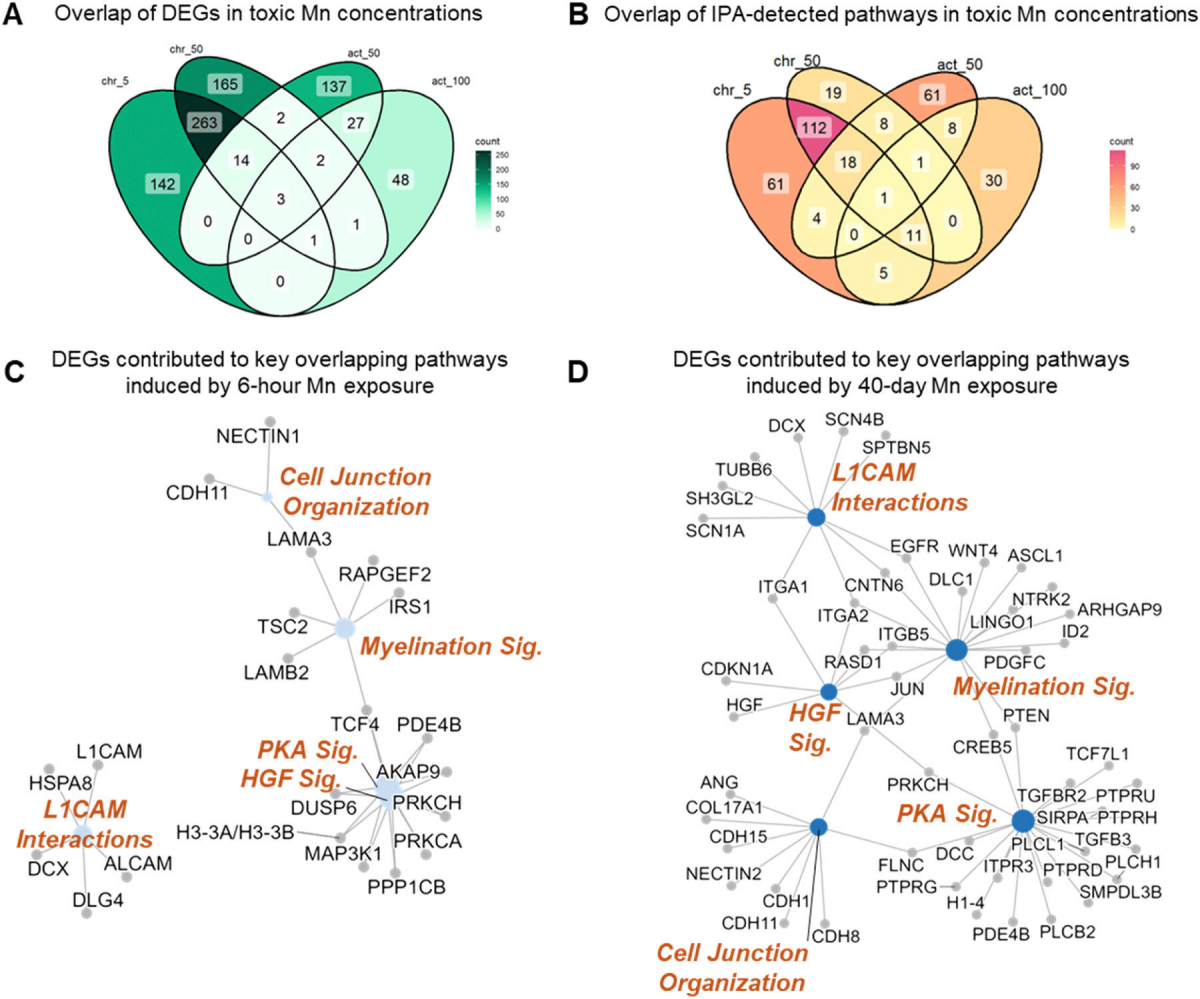
Overlap of genes and pathways significantly affected by both acute and chronic exposures. (A) Venn diagram of overlapping differentially expressed genes (DEGs) in 6-hour 50 μM (act_50), 6-hour 100 μM (act_100), 40-day 5 μM (chr_5), and 40-day 50 μM (chr_50). Numbers indicate the number of overlaps at the intersection. (B) Venn diagram of overlapping Ingenuity Pathway Analysis (IPA)-detected pathways in 6-hour 50 μM (act_50), 6-hour 100 μM (act_100), 40-day 5 μM (chr_5), and 40-day 50 μM (chr_50). Numbers indicate the number of overlaps at the intersection. (C) and (D) Cnet plots illustrating DEGs contributing to key pathways that are shared between acute (C) and chronic (D) exposures.

Our data provides evidence that toxic mechanisms under chronic conditions are distinct and could be distinguished from acute toxicity mechanisms. By plotting Venn diagrams of DEGs and IPA detected pathways affected by 6-hour 50 μM, 6-hour 100 μM, 40-day 5 μM, and 40-day 50 μM, the limited overlap supported this dissimilarity (Fig. 5A and B). This lack of shared DEGs and pathways between acute and chronic exposure supports a hypothesis that chronic Mn toxicity acts through distinct patterns and mechanisms that can only be partially represented by high concentrations of Mn exposures in acute duration experimental designs.

The only pathway that was shared across all selected conditions was the protein kinase A (PKA) signaling. However, DEGs that were altered in the PKA signaling were duration specific. *DUSP6* and *TCF4* were specifically affected by 6-hour 50 and 100 μM exposures, while *CREB5*, *DCC*, *FLNC*, *ITPR3*, *PDE4B*, *PRKCH*, and *TGFBR2* were only affected by 40-day 5 and 50 μM exposures. We examined the overlap of genes contributing to 48 pathways that were shared between acute and chronic exposures despite the Mn concentration by hypergeometric test (Supp. Table 3). Hypergeometric tests yielded an overall p value greater than 0.5 which confirmed no significant overlap when comparing DEGs contributing to the same shared pathway induced by 6-hour exposures and induced by 40-day exposures. Furthermore, selected key shared pathways and associated genes were illustrated in Fig. 5C and D, which displays that chronic exposures, although at lower concentrations, resulted in more gene expression changes and more connections amongst signaling pathways associated with the DEGs. This discordance between the DEGs detected in acute and chronic exposures add further support to the hypothesis that acute and chronic Mn toxicity act through distinct pathways and suggest that cellular responses to acute Mn insults at best only weakly represent chronic toxicity mechanisms.

### 3.6. Chronic exposures to Mn showed higher similarities to Parkinson’s disease transcriptomic data

To further evaluate the impact of exposure duration on the translational power of the transcriptomic outcomes, fold changes of DEGs detected from the present study were compared to publicly available datasets. Considering the similarity of symptoms between Mn-induced neurotoxicity and PD, two independent studies were incorporated. First, aligned counts were acquired from GSE206308 and differential expression analysis was performed as indicated by Verma et al. (Verma et al., 2023). This dataset was derived from human brain substantia nigra pars compacta (SNpc) from control individuals and PD patients. The other dataset was acquired from DEGs reported by Fiore et al. contrasting RA differentiated SH-SY5Y treated with PD-related neurotoxin 1-methyl-4-phenylpyridinium (MPP) and non-treated control (Fiore et al., 2022).

**Fig. 6.**
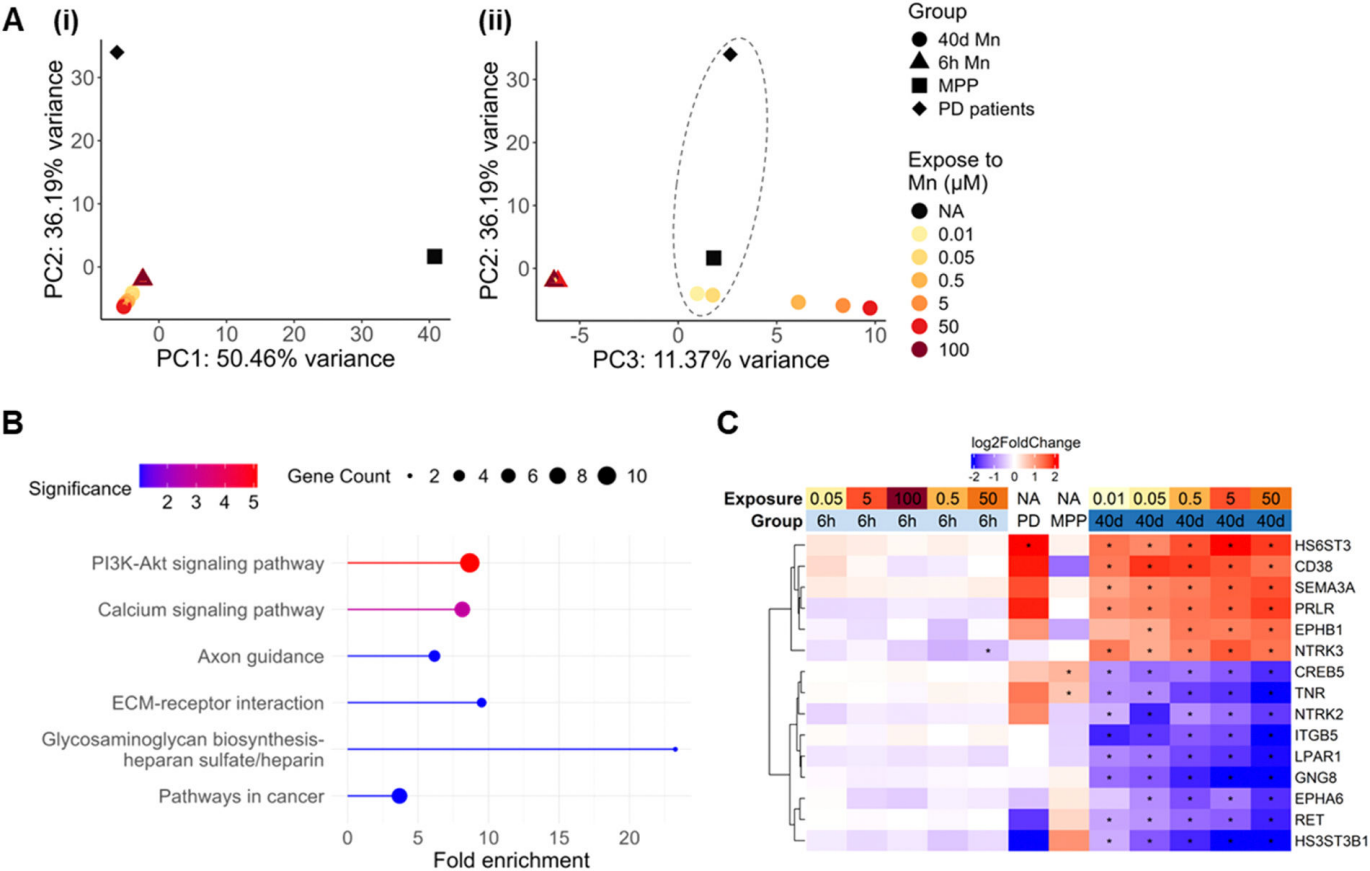
Comparison of differentially expressed genes detected in independent datasets. (A) Principal component analysis of fold changes differentially expressed genes (DEGs) detected in 40-day Mn exposure (40d Mn), 6-hour Mn exposure (6 h Mn), 1-methyl-4-phenylpyridinium-treated SH-SY5Y (MPP), and Parkinson’s disease patient postmortem brain (PD patients) RNA sequencing. (B) KEGG pathways that were significantly enriched (false discovery rate, FDR < 0.1) by key genes that contributed to the separation of samples along PC3 in Fig. 6A(ii). (C) Fold changes of genes that enrich pathways in Fig. 6B.

PCA analysis was first performed with the fold changes of detected DEGs in each dataset. As displayed in Fig. 6A(i), the major source of variation can be attributed to the source of dataset. Despite that, on PC3, which explained ~11 % of the total variance, 40-day Mn exposure at low concentrations (0.01 and 0.05 μM) clustered closer to both the PD patient and the MPP-treated SH-SY5Y dataset (Fig. 6A(ii)) compared to other Mn exposures. Therefore, it suggests that on PC3, 0.01 and 0.05 μM Mn exposed for 40 days had stronger correlations with PD-related transcriptomic changes. Based on this result, genes that contributed to the separation along PC3 were identified. Fig. 6B illustrated KEGG pathways that were significantly (false discover rate, FDR < 0.1) enriched by genes driving PC3 separation. These pathways included PI3K/AKT, calcium signaling, axon guidance, and extracellular matrix (ECM)-receptor interaction which were significantly or specifically detected in the 40-day exposure. Fold changes of genes that contributed to the significant enriched pathways were displayed in Fig. 6C. The correlation between PD-related and extended low-concentration Mn exposure-induced pathway changes again supported the hypothesis that chronic but not acute Mn exposures can reflect cellular alterations that are translatable to human neurotoxicity in a more comprehensive way.

## 4. Discussion

The present study contrasted transcriptomic changes induced by short and long-term Mn exposure with the widely used human neuroblastoma SH-SY5Y model. We aim to take advantage of the proliferation capability and methodological simplicity for culturing the SH-SY5Y cell model, while acknowledging its drawbacks in being an immortalized line. Therefore, by differentiating with RA and conditioning into human iPSC-derived neuron compatible culture media, we anticipate results from this study to initiate fast turn-around transcriptomic findings thus establish a translational bridge for future in-depth mechanistic investigations with complicated hiPSC-derived neuronal models.

Transcriptomic analysis performed in this study revealed distinct gene expression changes that contributed to dose and duration-dependent alterations. PCA plot displayed Fig. 4A which demonstrated differential effects of near-threshold (0.01 – 0.5 μM) versus toxic (50 and 100 μM) Mn concentrations agreed with prior findings with acute exposures where transcriptomic changes induced by 50 and 100 μM Mn were also distinguished from lower concentrations (Fernandes et al., 2019). Our analysis of concentration-dependent transcriptomic highlighted the upregulation of *TFRC* and *FTH1* also correlated with Fernandes’ findings where TF was identified as a key gene contributing dose-dependent effects (Fernandes et al., 2019). Investigation of distinguishable effects of acute and chronic exposure durations suggested that extended exposures had broader transcriptomic impact and stronger linkage to neurodegeneration pathology, represented by larger number of DEGs and enriched pathways with lower false discovery rate. These pathways include but were not limited to synapse assembly, cell junction assembly, and synaptic signaling (Fig. 3C). Pathways that were correlated with Mn responsive genes can be clustered into two leading groups: metallic ion transportation and homeostatic regulation, and enzymatic cascades that utilize Mn as a cofactor under physiological conditions.

The involvement of metallic ion homeostatic maintenance system in Mn-induced neurotoxicity has been reported in various modeling systems (Aschner et al., 2007; Balachandran et al., 2020; Yokel, 2006). Amongst genes that display Mn concentration-dependent responses highlighted in Fig. 4B, *TFRC*, *FTH1*, and *YIPF7* were direct or potential Mn homeostasis regulators. *TFRC* (transferrin receptor), which was significantly enhanced by Mn concentrations beyond 0.5 μM regardless of exposure duration, encodes transferrin receptor 1 (TfR1) that contributes partially to neuronal uptake of both Mn^2+^ and Mn^3+^ and the iron-Mn homeostasis regulation (Gunter et al., 2013; Q. Liu et al., 2021; Xin et al., 2017). The upregulation of *TFRC* mRNA and TfR1 has been reported in brain and liver responses to Mn exposures (Chen et al., 2024; Pettiglio et al., 2016). *FTH1* (ferritin heavy chain 1) was downregulated by low concentrations of Mn and upregulated by toxic Mn in our results. A similar U-shape dose dependent *FTH1* expression change was reported in rhesus monkey liver exposed to inhaled Mn for 65 days (Pettiglio et al., 2016). Altered expressions of *TFRC* and *FTH1* significantly mapped with ferroptosis (ShinyGO KEGG pathway analysis, FDR = 0.0025) which has been reported as a key cytotoxic mechanism in high dose Mn exposures (Z. Zhang et al., 2025). *YIPF7* encodes member 7 of the YIP domain family, which is a family of transmembrane proteins that were predicted to localize in the Golgi apparatus (Shaik et al., 2019). Despite lack of comprehensive characterization of *YIPF7* function, the localization of YIPF7 on Golgi, which is an organelle that acts as an essential intracellular Mn sink and holds abundant Mn and metallic ion transporters, implicates a potential regulatory role of YIPF7 in intracellular Mn distribution (Carmona et al., 2010; Van Baelen et al., 2004).

Besides metal transporter encoding genes that were detected in Mn concentration-dependent gene expression changes, IPA ‘metallothioneins bind metals’ pathway was significantly affected at neurotoxic Mn concentrations (50 μM and above) in both 6-hour and 40-day exposures. Despite controversy reports regarding whether metallothionein (MT) genes and/or proteins expression levels are potentiated or inhibited by Mn exposure, there is profound evidence that MTs are responsive to Mn accumulation in the central nervous system and perturbed MT expression is linked with oxidative stress, inflammation, and mitochondrial dysfunction thus contributing to neurodegeneration induced by Mn intoxications (Baesler et al., 2021; Erikson and Aschner, 2002; Forcella et al., 2020; Mezzaroba et al., 2019; Miyazaki and Asanuma, 2023; Nishito et al., 2024; Penkowa and Hidalgo, 2001). Although actively responsible for neurotoxicity induced by elevated intracellular Mn, MTs do not directly bind to Mn ion but to other divalent metals such as zinc, copper, chromium, cadmium, and mercury (Kobayashi et al., 2007). Therefore, the altered MT expression hints that Mn exposures may also disturb homeostasis of other metals. Mn accumulation in the brain has been confirmed to affect zinc, chromium, and iron levels, while the fluctuations of the bioavailability of these metals in turn leads to affect MT expression levels (Chung et al., 2010; Kim and Kang, 2016; Lee and Koh, 2010; Miah et al., 2020; Peterson and Fujinami, 2007).

These findings validate the mechanistic role of metallic ion homeostasis regulation in Mn-induced neurotoxicity. Furthermore, future investigations of genetic intracellular Mn content regulators, in combination with other genes identified in Fig. 4B could potentially serve as preliminary starting points for benchmarking *in vitro* Mn neurotoxicity deviation concentration. The expression patterns of these genes can be measured to distinguish whether the designated exposure triggers subtoxic adaptations or toxic adverse response, thus overcoming the challenges of assessing environmentally and human brain relevant external Mn concentrations in cell-based models. Furthermore, the metallic transporter and binding proteins we identified interact not only with Mn but iron, zinc, and calcium. This crosstalk implies that elevated intracellular Mn can lead to interruptions of overall cellular metallic content homeostasis. It also points towards future directions of characterizing Mn impact on the kinetics of other metals, which has been reported in epidemiology and animal studies but not fully understood (Criswell et al., 2015; Guilarte et al., 2006; Mercadante et al., 2016). Comprehensive understanding of the intracellular metallic homeostasis would facilitate further understanding of brain responses to environmental Mn insults.

In addition to intracellular metal content regulation that was discussed above, pathways in which protein/ enzymatic components utilize Mn as a cofactor under physiological conditions were also significantly impacted by Mn exposures, especially under chronic conditions. The complicated role of Mn-dependent biology and its neurotoxicity have been covered in prior literature reviews from our lab (Balachandran et al., 2020; Pfalzer and Bowman, 2017). Here in this study, we identified CREB, PKA, S100 family, and STAT3 signaling to be impacted from the lowest to highest ends of applied Mn chronic concentrations (Fig. 2D). PKA activation by 3′−5′-cyclic adenosine monophosphate (cAMP) phosphorylates downstream transcription factor cAMP response element binding protein (CREB) (Sadeghian et al., 2022). In the central nervous system, cAMP/PKA/CREB cascade is responsible for promoting neuron survival, modulating synaptic plasticity, and strengthening synaptic transmission efficiency (Kandel, 2012; J. Kim and Kaang, 2023). Mn is involved in the fine tuning of PKA kinase activity by enhancing the binding of PKA to ADP, amino acids, and its substrates (Knape et al., 2017). Moreover, the activation of CREB modulates the gene expression of manganese superoxide dismutase (MnSOD) which is in turn a reflection of Mn bioavailability (Kim et al., 1999; Luo et al., 2007). Transcriptomic alterations observed in our results align with prior findings on protein phosphorylation levels in other experimental models. Increased phosphorylation of CREB was reported in PC12 rat neuronal line exposed to 300 μM for 24 hours and 500 μM for 3 and 6 hours, as well as mice exposed to 20 mg/kg body weight for 60 consecutive days (Zhu et al., 2019). Extension of the exposure may potentially lead to a reversal effect that downregulates the CREB phosphorylation, as it was reported in PC12 exposed to 500 μM for 24 hours and rats that were exposed to 5 – 20 mg Mn/kg body weight for 126 days (Liang et al., 2015). Decreased CREB gene and protein expression were also reported consequences of developmental exposures (Wang et al., 2017). The contradictory regulation of PKA/CREB signaling activity aligns with our findings and provides a good example of the biphasic response of Mn-dependent biology when intracellular Mn content is elevated.

The PKA/CREB signaling was also one of the key targets shared between acute and chronic exposures. In addition, extracellular matrix organization and cellular morphological and junction regulation were amongst the most affected pathways in both durations. The Mn impact on these pathways was partially driven by gene expression changes in integrin subunits α1 (*ITGA1*) and β5 (*ITGB5*) that were detected in chronic exposures. Integrins are a family of cell adhesion proteins for which binding to their ligand requires Mn; enhanced synthesis of integrin alpha subunits promoted PC12 neurite outgrowth treated with low concentration Mn (10 μM) (Lein et al., 2000). Enrichment of neurite and axonal growth genes were also reported in *slc39a14*^−/−^ induced Mn deficiency in zebrafish supporting that normal functioning of this pathway requires optimal Mn supplementation (Tuschl et al., 2022).

As discussed above, perturbances in metallic homeostasis regulation and Mn-dependent biological processes are key cellular responses in both 6-hour and 40-day Mn overloads. However, 40-day exposures led to more complicated transcriptomic alterations when compared to changes induced by 6-hour exposures. It was displayed in Fig. 2B that genes contributing to the main variance between 40-day and 6-hour exposures had increasing fold changes with the elevation of Mn concentrations in chronic exposures. Figs. 5C and 5D demonstrated that in 40-day exposures, there were more DEGs contributing to the same pathway that were detected in 6-hour exposures. Furthermore, extended exposures led to duration-specific enrichment in axonal guidance signaling and amyloid fiber formation pathways. Leading genes that contributed to the enrichment of axonal guidance and amyloid fiber formation pathways, including *CALB1* (calcium-buffering protein calbindin), *PRL* (prolactin, whose expression is restricted by dopamine), *NPPA* (natriuretic peptide precursor A), and *DCC* (netrin-1 receptor), regulate neuronal connectivity and plasticity, thus contribute to cognitive functions and neurodegenerative disease pathology (Datta et al., 2024; Horn et al., 2013; Keo et al., 2021; Kim et al., 2009; Minaya et al., 2023). It was concluded in a genome-wide association study (GWAS) that variations in axonal guidance pathway can be a predictive marker for PD outcomes (Lin et al., 2009). Amyloid fiber formation pathway was reported in animal model-based and patient-based transcriptomic studies to be strongly correlated to Alzheimer’s and Parkinson’s disease (Bandres-Ciga et al., 2020; Lamisa et al., 2024; Liu et al., 2024). Beyond highlighting disease-related pathways, Fig. 6 evaluation of DEGs detected in Mn exposures, MPP-induced SH-SY5Y PD model, and PD patients revealed that 40-day Mn treatment at low concentrations (0.01 and 0.05 μM) displayed the highest similarity to PD-related transcriptomic changes. The resemblance in gene expression alterations across these independent datasets reaffirmed PD risks reported in long-term environmental and occupational Mn exposed populations (Kwakye et al., 2015; Racette et al., 2021). Therefore, it implies that extended time frame of the exposure can more effectively simulate transcriptomic alterations associated with human neurotoxicity outcomes. However, although the comparison across independent datasets highlighted chronic-specific axon guidance and ECM-receptor pathways which supported the significance of the extended time frame, some discordances were observed in the directionality of the DEGs (Fig. 6C). Further investigations with currently incorporated datasets and inclusion of a wider range of neurodegenerative disease transcriptomic data would provide future insights for the translational power of chronic sub-cytotoxic design in SH-SY5Y models and the correlation between Mn exposures and later-life neurodegeneration as well.

The detection of genes and pathways specifically in chronic exposures confirms that extending the exposure duration not only led to more DEGs with higher fold changes but reveals mechanisms that are more closely related to neuronal functions and better simulate the epidemiology evidenced link between Mn exposures and neurodegeneration. Moreover, extended exposures led to gene expression changes that interconnect with alterations detected under acute conditions. The metalloprotease-disintegrin (ADAM) family genes that predominantly contributed to the chronic exposure-specific alterations in axonal guidance signaling pathway supports integrin-mediated extracellular matrix organization and cell adhesion that were affected by both acute and chronic exposures. MT expression, which was significantly stimulated when Mn concentration went beyond 50 μM, is activated through interleukin-6 (IL-6) mediated JAK/STAT3 signaling which was detected in chronic exposures at lower concentrations (Jamrozik et al., 2023; Liang et al., 2016; Mayor-Ibarguren et al., 2020). Both ADAM family and JAK/STAT3 signaling, in the meantime, act as coordinators of brain amyloid precursor protein expression thus entwine with the amyloid fiber formation pathway that was significantly affected solely by 40-day exposures (Myhre et al., 2013; Yang et al., 2006). Collectively, investigations contrasting transcriptomic changes induced by acute and chronic exposure indicate that extending the exposure duration from 6 hours to 40 days led to increased magnitude and scope of DEGs as well as developed cross-talking networks that strongly affirm the neurodegenerative risk that long-term Mn exposure could impose in humans.

In conclusion, this study highlighted the distinguishable impact of chronic near-threshold Mn exposure on the differentiated SH-SY5Y transcriptome. We provided evidence to support the hypothesis that acute exposures at high concentrations weakly represent long-term near-threshold exposures. Compared to acute experimental settings, extended durations led to transcriptomic changes that more profoundly connect to pathways implicated in neurodegenerative disease pathology. Beyond capturing specific pathways associated with chronic Mn-induced central nervous system disorders, prolonged exposures led to more complicated gene expression changes even when comparing within pathways that were shared between durations. In addition, we identified potential genetic markers that could differentiate acute toxic conditions from chronic near-threshold levels. These markers are of future beneficial to the field by proposing a novel “bottom-up” approach to determine environmentally and human exposure relevant *in vitro* paradigms, while avoiding the undefined brain extracellular physiological and post-exposure Mn levels. These findings not only enhanced our understanding of the role of Mn-dependent biology in its neurotoxicity, but also set the stage for future mechanistic studies to decipher human exposure and disease risks associated Mn adverse outcome pathways via *in vitro* modeling.

## Supplementary Material

MMC2

MMC1

## Figures and Tables

**Table 1:** Qiagen Ingenuity Pathway Analysis (IPA) of 76 genes that were differentially expressed across all 40-day exposure concentrations.

Ingenuity Canonical Pathways	−log(p-value)	Ratio	z-score	Molecules
Extracellular matrix organization	4.77	0.0472	−2.236	ITGA1,ITGB5,LRP4, NID1,TNR
Axonal Guidance Signaling	4.66	0.0176	NA	ADAMTS2,ITGA1,ITGB5,LRRC4C,NTNG1,NTRK3,PGF,PLCL1,SEMA3A
RAR Activation	3.51	0.0163	−1.134	CRABP2,CREB5,DIRAS3,DKK1,DKK2,RET,TGFBR2
PTEN Signaling	2.95	0.0265	NA	ITGA1,ITGB5,NTRK3,TGFBR2
Factors Promoting Cardiogenesis in Vertebrates	2.93	0.0261	1	CREB5,DKK1,PLCL1,TGFBR2
CREB Signaling in Neurons	2.64	0.0115	1.134	CREB5,GIPR,LPAR1,NTRK3,PLCL1,PTGER3,TGFBR2
Actin Nucleation by ARP-WASP Complex	2.55	0.0323	NA	DIRAS3,ITGA1,ITGB5
Effects of PIP2 hydrolysis	2.53	0.0741	NA	DGKB,RASGRP1
TCF dependent signaling in response to WNT	2.51	0.0201	−1	DACT1,DKK1,DKK2,H2AC6
ILK Signaling	2.5	0.0199	0	CREB5,DIRAS3,ITGB5,PGF
Ephrin Receptor Signaling	2.49	0.0198	NA	CREB5,ITGA1,ITGB5,PGF
Molecular Mechanisms of Cancer	2.39	0.00935	0.707	DIRAS3,GIPR,ITGA1,ITGB5,LPAR1,PTGER3,RASGRP1,TGFBR2
RHOGDI Signaling	2.36	0.0182	NA	CDH11,DIRAS3,ITGA1,ITGB5
Regulation of Actin-based Motility by Rho	2.3	0.0261	NA	DIRAS3,ITGA1,ITGB5
ROBO SLIT Signaling Pathway	2.18	0.0236	NA	CNTNAP2,PGF,TGFBR2
Signaling by Rho Family GTPases	2.07	0.015	NA	CDH11,DIRAS3,ITGA1,ITGB5
Insulin Secretion Signaling Pathway	2.02	0.0145	1	CREB5,GIPR,PLCL1,PRLR
Dermatan Sulfate Biosynthesis (Late Stages)	2	0.04	NA	CHST15,HS6ST3

Differentially expressed genes (DGEs) were defined based on statistic cutoff of fold change over 1.5 and adjusted p value less than 0.1. Identified pathways that showed a p value less than 0.01 (−log(p-value) > 2) were displayed. Ratio was calculated based on the number of molecules in a given pathway, divided by total number of molecules that make up that pathway and that are in the Ingenuity genes only knowledge base reference set. The z-scores reflect a predicted activation (≥ 2) or inhibition (≤ −2) of a given pathway based on comparing the fold change of molecules with the pattern that is expected based on the literature. NAs in z-scores indicate that there were not enough number of genes associated with a particular pathway to provide any reliable predictions.

## Data Availability

Data will be made available on request.

## Appendix A. Supporting information

Supplementary data associated with this article can be found in the online version at doi:10.1016/j.neuro.2026.103393.
