# Supplementary material for: Prolonged duration induces divergent transcriptomic responses to manganese, distinct from concentration effects, in an SH-SY5Y neurotoxicity model
Source: Neurotoxicology. Author manuscript; Available in PMC 2026 May 4. (PMC13138507; doi:10.1016/j.neuro.2026.103393)
Supplement: MMC1 [file NIHMS2144622-supplement-MMC1.docx]

1. **Supplemental materials**


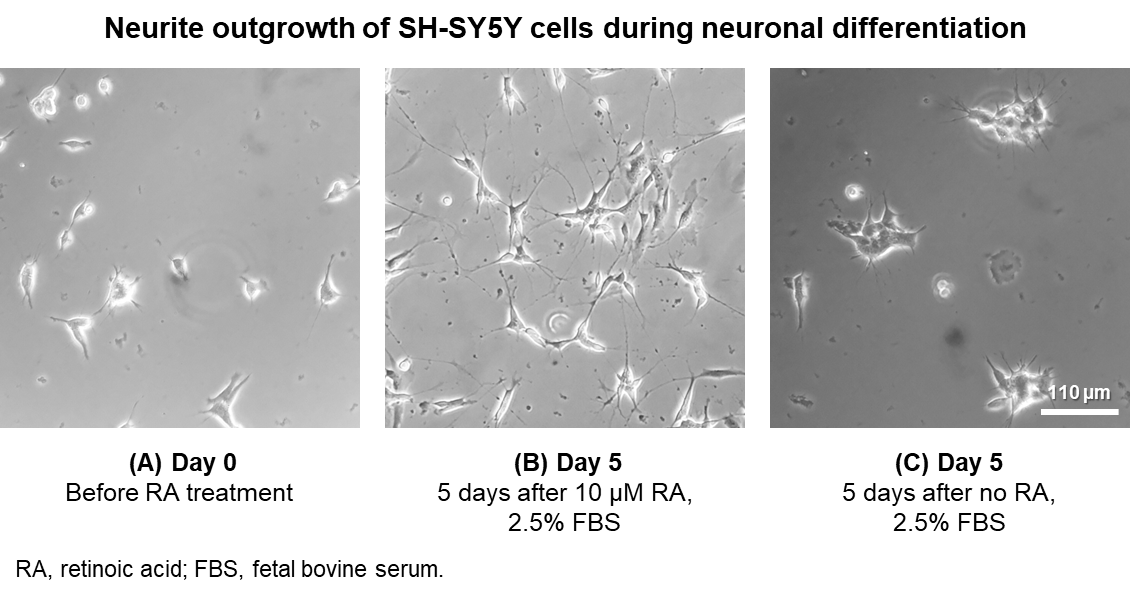


**Supplemental Figure 1** Brightfield images of SH-SY5Y cells on day 0 (A) and day 5 (B and C) of neuronal differentiation. RA, retinoic acid, FBS, fetal bovine serum.


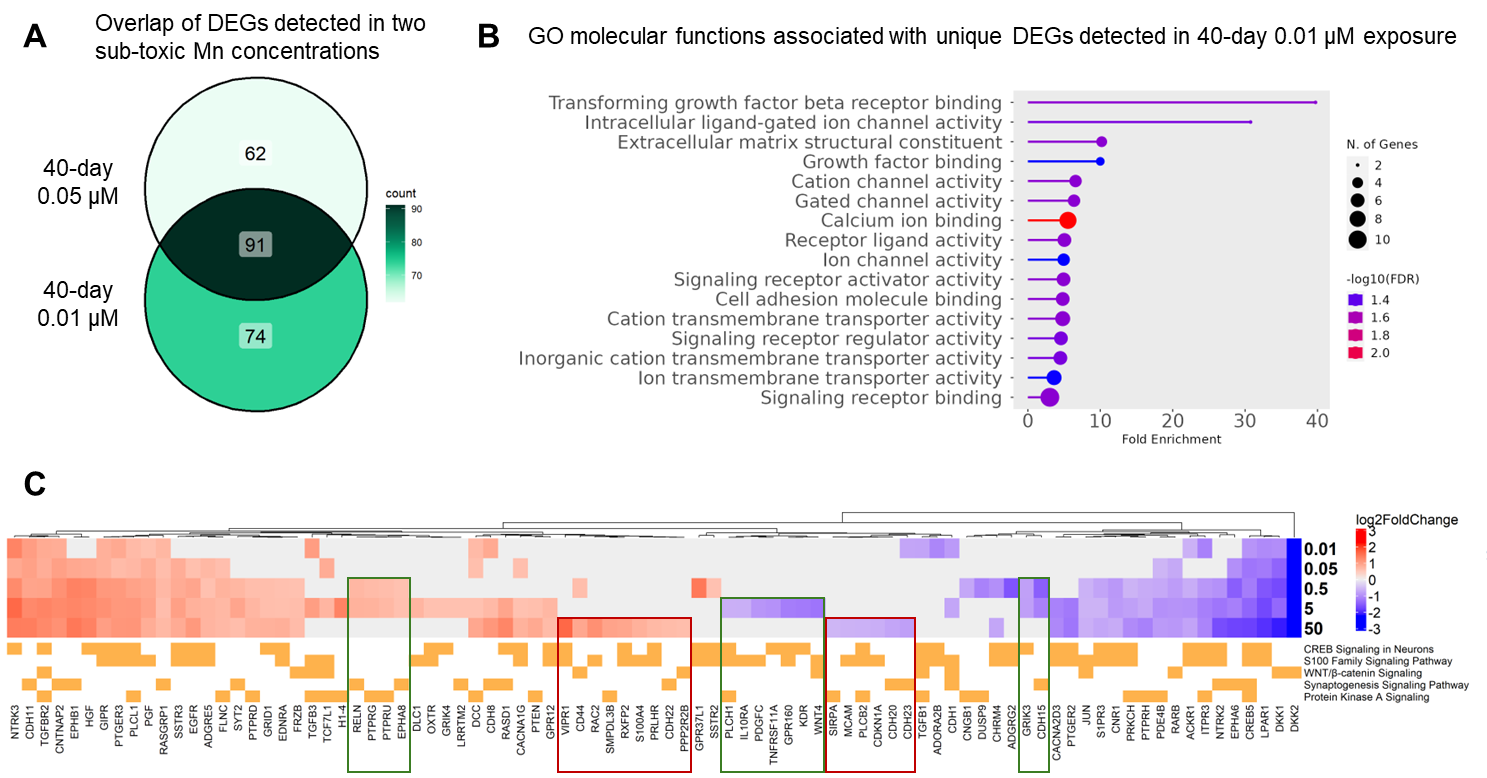


**Supplemental Figure 2** Dose-dependent gene expression changes and associated pathways in 40-day exposure

**(A)** Overlap of differentially expressed genes (DEGs) detected in 40-day 0.01 µM and 40-day 0.05 µM exposures. DEGs determined with a cutoff of absolute fold change over 1.5 and adjusted p value less than 0.1. Numbers indicate number of genes at each intersection. **(B)** Enrichment analysis mapping DEGs detected only 40-day 0.01 µM but not 0.05 µM exposure to GO molecular functions database. Analysis performed by ShinyGO 0.80 with a false discovery rate (FDR) cutoff of 0.05. **(C)** Heatmap clustering of fold changes in DEGs that contribute to the detection of selected pathways in Figure 2D that are shared across all chronically exposed Mn concentrations. Row names marked the concentration of Mn. Column annotation labeled genes that are associated with a particular pathway in orange. DEGs that are only detected in 50 µM exposure are highlighted in red boxes, while DEGs that are detected in 0.5 and/or 5 µM but not 50 µM are highlighted in green boxes.


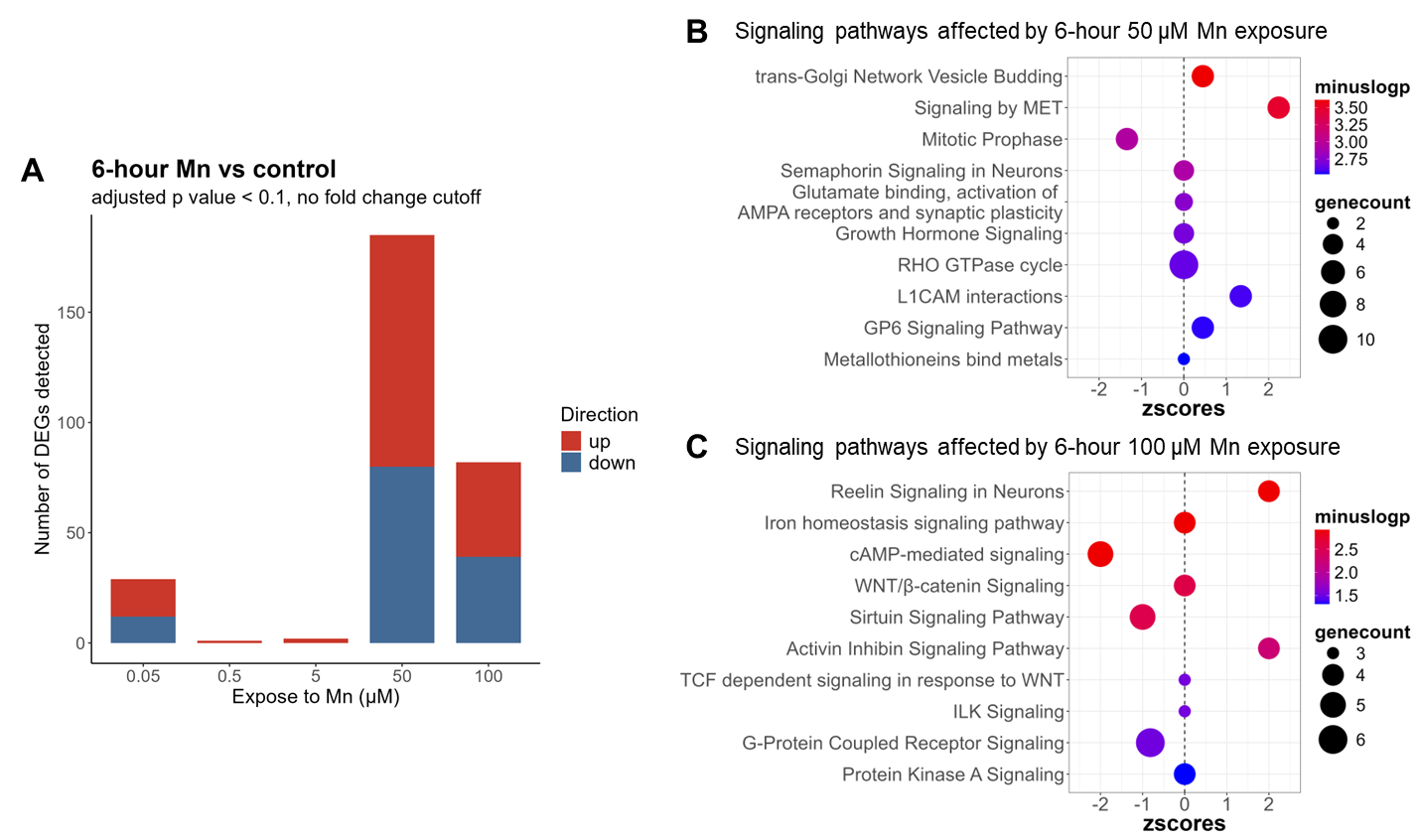


**Supplemental Figure 3** Gene expression changes in 6-hour Mn exposures. **(A)** Number of differentially expressed genes (DEGs) identified with adjusted p value cutoff of 0.1 contrasting each exposure concentration to non-exposed control. **(B)** and **(C)** Signaling pathways associated with DEGs detected in 6-hour 50 µM (B) and 6-hour 100 µM (C) by Qiagen Ingenuity Pathway Analysis (IPA). Dots were color coded with -log(p-value) in which higher values indicate greater significance. The sizes of the dots represent the number of DEGs that contributed to the detection of a particular pathway. Z-scores inform predicted activation (positive) or inhibition (negative) of a particular pathway. A z-score of zero indicates that there was either no sufficient agreement between expression patterns detected in the current dataset and available literature, or not enough number of genes contributing to the pathway.


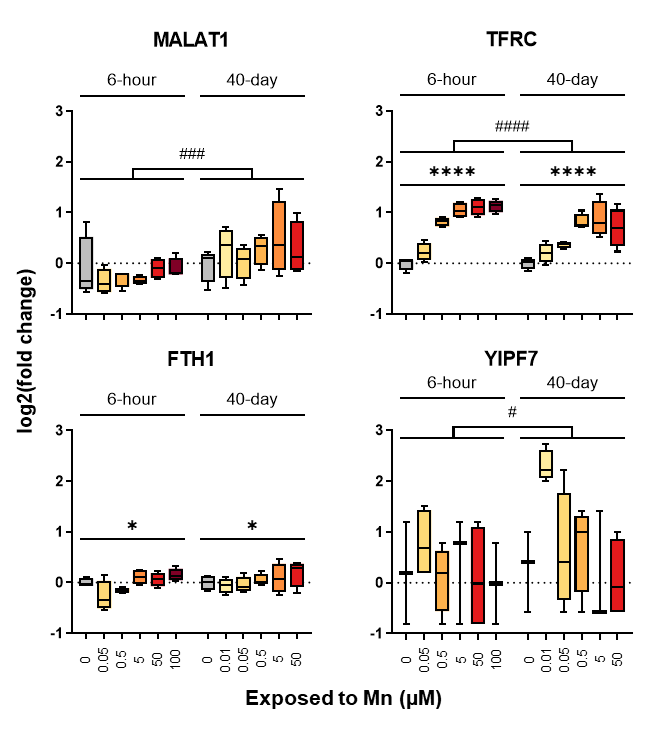


**Supplemental Figure 4** Fold change of genes that holds the potential to be markers distinguishing exposure durations (MALAT1) or levels (TFRC, FTH1, YIFP7). Log2(fold change) was calculated by log2(Counts_treated_/Average Counts_control_). * *P* < 0.05, *** *P* < 0.01, **** *P* < 0.0001 indicated significant variation across different Mn concentrations determined by RM-two-way ANOVA. # *P* < 0.05, ### *P* < 0.01, #### *P* < 0.0001 indicated significant variation between 6-hour and 40-day exposure duration determined by RM-two-way ANOVA.


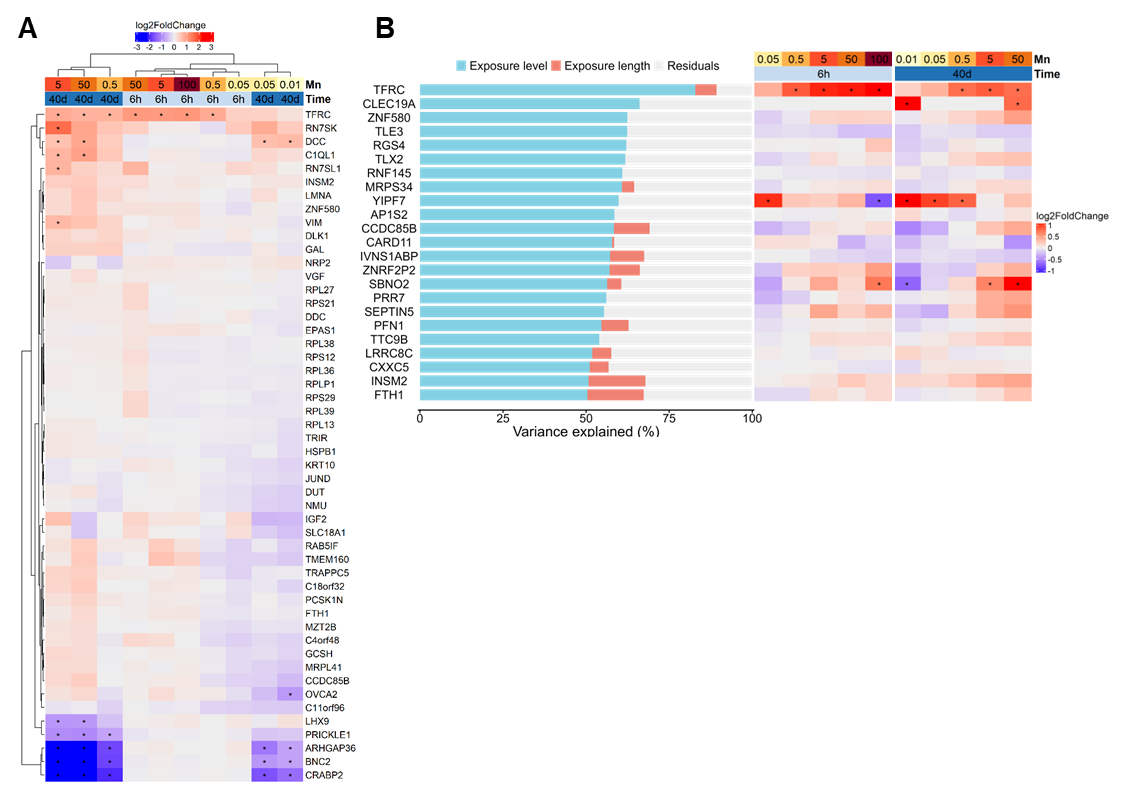


**Supplemental Figure 5** Genes in which counts variance are mainly contributed by increasing Mn concentrations. **(A)** Expression pattern of 50 genes that show highest loading values in principal component 2 in the principal component analysis in Figure 3A/4A. **(B)** Percentage variance explained and expression pattern of genes in which over 50% counts variance are attributed to Mn exposure levels determined by variance partitioning analysis.
